# 1,3-Diallyl-6-bromo-1*H*-imidazo[4,5-*b*]pyridin-2(3*H*)-one

**DOI:** 10.1107/S1600536811026869

**Published:** 2011-07-09

**Authors:** Siham Dahmani, Youssef Kandri Rodi, Santiago V. Luis, Michael Bolte, Lahcen El Ammari

**Affiliations:** a Laboratoire de Chimie Organique Appliquée, Université Sidi Mohamed, Ben Abdallah, Faculté des Sciences et Techniques, Route d’Immouzzer, BP 2202 Fès, Morocco; b Departamento de Quimica Inorganica & Organica, ESTCE, Universitat Jaume I, E-12080 Castellon, Spain; c Institut für Anorganische Chemie, J. W. Goethe-Universität Frankfurt, Max-von-Laue-Strasse 7, 60438 Frankfurt/Main, Germany; d Laboratoire de Chimie du Solide Appliquée, Faculté des Sciences, Université Mohammed V-Agdal, Avenue Ibn Battouta, BP 1014, Rabat, Morocco

## Abstract

In the mol­ecule of the title compound, C_12_H_12_BrN_3_O, the fused-ring system is essentially planar, the largest deviation from the mean plane being 0.0148 (3) Å. The two allyl groups are nearly perpendicular to the imidazo[4,5-*b*]pyridine plane [C—C—N—C torsion angles of 81.6 (4) and −77.2 (4)°] and point in the same direction. The planes through the atoms forming each allyl group are nearly perpendicular to the imidazo[4,5-*b*]pyridin-2-one system, as indicated by the dihedral angles between them of 80.8 (5) and 73.6 (5)°.

## Related literature

For background to the biological activity of substituted imidazopyridines and related compounds, see: Barraclough *et al.* (1990 ▶); Bavetsias *et al.* (2007 ▶, 2010 ▶); Coates *et al.* (1993 ▶); Liu *et al.* (2008 ▶); Ryabukhin *et al.* (2006 ▶); Schiffmann *et al.* (2006 ▶).
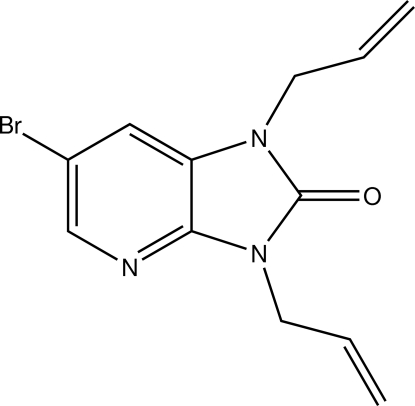


## Experimental

### 

#### Crystal data

C_12_H_12_BrN_3_O
*M* *_r_* = 294.16
Orthorhombic, 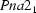

*a* = 5.4110 (3) Å
*b* = 25.4205 (12) Å
*c* = 9.3170 (4) Å
*V* = 1281.56 (11) Å^3^
*Z* = 4
Mo *K*α radiation
μ = 3.20 mm^−1^
*T* = 273 K
0.52 × 0.32 × 0.14 mm

#### Data collection

Bruker CCD three-circle diffractometer
Absorption correction: multi-scan (*SADABS*; Bruker, 1997 ▶) *T*_min_ = 0.202, *T*_max_ = 0.800
8789 measured reflections
3201 independent reflections
2361 reflections with *I* > 2σ(*I*)
*R*_int_ = 0.027

#### Refinement

*R*[*F* ^2^ > 2σ(*F* ^2^)] = 0.036
*wR*(*F* ^2^) = 0.100
*S* = 1.03
3201 reflections
156 parameters
1 restraint
H-atom parameters constrained
Δρ_max_ = 0.44 e Å^−3^
Δρ_min_ = −0.23 e Å^−3^
Absolute structure: Flack (1983) ▶, 1494 Friedel pairs
Flack parameter: 0.040 (17)

### 

Data collection: *SMART* (Bruker, 1997 ▶); cell refinement: *SAINT* (Bruker, 1997 ▶); data reduction: *SAINT*; program(s) used to solve structure: *SHELXS97* (Sheldrick, 2008 ▶); program(s) used to refine structure: *SHELXL97* (Sheldrick, 2008 ▶); molecular graphics: *ORTEP-3 for Windows* (Farrugia, 1997 ▶); software used to prepare material for publication: *WinGX* (Farrugia, 1999 ▶).

## Supplementary Material

Crystal structure: contains datablock(s) I, global. DOI: 10.1107/S1600536811026869/om2445sup1.cif

Structure factors: contains datablock(s) I. DOI: 10.1107/S1600536811026869/om2445Isup2.hkl

Supplementary material file. DOI: 10.1107/S1600536811026869/om2445Isup3.cml

Additional supplementary materials:  crystallographic information; 3D view; checkCIF report

## Figures and Tables

**Fig. 1.**
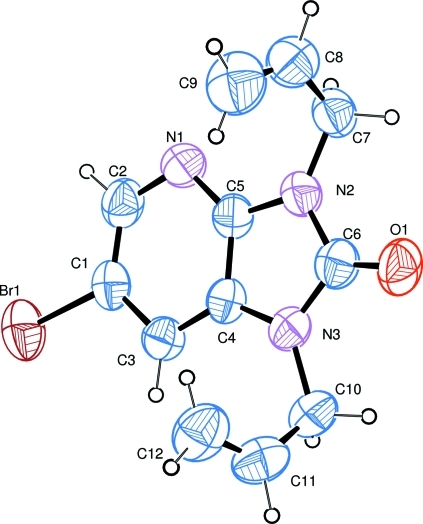
: Plot of the molecule of the title compound with the atom-labelling scheme. Displacement ellipsoids are drawn at the 50% probability level. H atoms are represented as small circles.

## supplementary crystallographic information

### Comment

Substituted imidazopyridines and structurally related compounds are of pharmacological and therapeutical interest. They have been tested for their potential as anticancer, inotropic (Barraclough *et al.*, 1990), selective antihistamine (H1) agents and antibacterial activity (Liu *et al.*, 2008). Imidazo[4,5-*b*]pyridine derivatives were also reported as Aurora kinases (Bavetsias *et al.*, 2007); Bavetsias *et al.*, 2010), and cyclic PDE inhibitors (Coates *et al.*, 1993). The preparation of these compounds is usually straightforward, and a number of synthetic methods are already available (Ryabukhin *et al.*, 2006; Schiffmann *et al.*, 2006).

In this work, we report the synthesis of 1,3-diallyl-6-bromo-1,3-dihydroimidazo [4,5-*b*]pyridin-2-one *via* the reaction between 6-bromo-1,3-dihydro-imidazo [4,5 - b-]pyridin-2-one and allylbromide in DMF using K_2_CO_3_ as base (scheme1).

The Plot of the title compound molecule is shown in Fig.1. The two fused five and six-membered rings are nearly planar with the maximum deviation of -0.014 (3)Å from N1. The two allyl chains (–C7–C8–C9) and (–C10–C11–C12) are almost perpendicular to the imidazo[4,5-*b*]pyridine system mean plane as indicated by the following torsion angles C8–C7–N2–C5 and C11–C10–N3–C4 of 81.6 (4)° and -77.2 (4) ° respectively.

### Experimental

To a stirred solution of 6-bromo-1,3-dihydro-imidazo[4,5 - b-]pyridin-2- one (0.5 g; 2.33 mmol), K_2_CO_3_ (1.29 g; 9.34 mmol), and tetrabutylammonium bromide (0.07 g; 2.37 10 ^-4^ mol) in DMF, allylbromide (0.5 ml; 5.84 mmol) was added dropwise. Stirring was continued at room temperature for 24 h. After completion of reaction (monitored by TLC), the salt was filtered and the solvent was removed under reduced pressure. The resulting residue was purified by column chromatography on silica gel using ethylacetate/hexane (1/1) as eluent. The crystals of the title compound are obtained by dissolving 80 mg of product in 4 ml of ethanol at about 353 K, followed by a slow evaporation of the solvent.

### Refinement

The origin of the non centro symmetric space group is fxed by the *SHELXL* program and the 1495 Friedel opposite refletions are not merged. H atoms were located in a difference map and treated as riding with C—H = 0.93 Å and 0.97 Å for aromatic and methylene respectively. All H atoms with *U*_iso_(H) = 1.2 *U*_eq_ (aromatic and methylene).

### Crystal data

**Table:**

C_12_H_12_BrN_3_O	*F*(000) = 592
*M**_r_* = 294.16	*D*_x_ = 1.525 Mg m^−^^3^
Orthorhombic, *P**n**a*2_1_	Mo *K*α radiation, λ = 0.71073 Å
Hall symbol: p 2c -2n	Cell parameters from 3201 reflections
*a* = 5.4110 (3) Å	θ = 1.6–28.5°
*b* = 25.4205 (12) Å	µ = 3.20 mm^−^^1^
*c* = 9.3170 (4) Å	*T* = 273 K
*V* = 1281.56 (11) Å^3^	Block, colourless
*Z* = 4	0.52 × 0.32 × 0.14 mm

### Data collection

**Table:**

Bruker CCD three-circle diffractometer	3201 independent reflections
Radiation source: sealed tube	2361 reflections with *I* > 2σ(*I*)
graphite	*R*_int_ = 0.027
phi and ω scans	θ_max_ = 28.5°, θ_min_ = 1.6°
Absorption correction: multi-scan (*SADABS*; Bruker, 1997)	*h* = −6→7
*T*_min_ = 0.202, *T*_max_ = 0.800	*k* = −25→34
8789 measured reflections	*l* = −12→12

### Refinement

**Table:**

Refinement on *F*^2^	Secondary atom site location: difference Fourier map
Least-squares matrix: full	Hydrogen site location: inferred from neighbouring sites
*R*[*F*^2^ > 2σ(*F*^2^)] = 0.036	H-atom parameters constrained
*wR*(*F*^2^) = 0.100	*w* = 1/[σ^2^(*F*_o_^2^) + (0.0402*P*)^2^ + 0.2176*P*] where *P* = (*F*_o_^2^ + 2*F*_c_^2^)/3
*S* = 1.03	(Δ/σ)_max_ < 0.001
3201 reflections	Δρ_max_ = 0.44 e Å^−^^3^
156 parameters	Δρ_min_ = −0.23 e Å^−^^3^
1 restraint	Absolute structure: Flack (1983), 1494 Friedel pairs
Primary atom site location: structure-invariant direct methods	Flack parameter: 0.040 (17)

### Special details

**Table:**

Geometry. All e.s.d.'s (except the e.s.d. in the dihedral angle between two l.s. planes) are estimated using the full covariance matrix. The cell e.s.d.'s are taken into account individually in the estimation of e.s.d.'s in distances, angles and torsion angles; correlations between e.s.d.'s in cell parameters are only used when they are defined by crystal symmetry. An approximate (isotropic) treatment of cell e.s.d.'s is used for estimating e.s.d.'s involving l.s. planes.
Refinement. Refinement of *F*^2^ against all reflections. The weighted Rw factor and goodness of fit *S* are based on *F*^2^, conventional *R*-factors *R* are based on *F*, with *F* set to zero for negative *F*^2^. The threshold expression of *F*^2^ > σ(*F*^2^) is used only for calculating *R*-factors(gt) *etc*. and is not relevant to the choice of reflections for refinement. *R*-factors based on *F*^2^ are statistically about twice as large as those based on *F*, and *R*- factors based on all data will be even larger.

### Fractional atomic coordinates and isotropic or equivalent isotropic displacement parameters (Å^2^)

**Table:**

	*x*	*y*	*z*	*U*_iso_*/*U*_eq_	
Br1	0.99838 (6)	0.148410 (15)	0.99886 (11)	0.07396 (14)	
N1	0.5607 (5)	0.19539 (10)	0.6617 (3)	0.0585 (6)	
N2	0.2257 (4)	0.14754 (9)	0.5520 (3)	0.0541 (6)	
N3	0.2480 (5)	0.07428 (9)	0.6813 (3)	0.0552 (6)	
O1	−0.0599 (4)	0.08103 (10)	0.5080 (4)	0.0730 (6)	
C1	0.7552 (5)	0.14820 (11)	0.8523 (3)	0.0515 (7)	
C2	0.7283 (6)	0.19139 (12)	0.7678 (4)	0.0592 (8)	
H2	0.8321	0.2199	0.7842	0.071*	
C3	0.6043 (6)	0.10361 (12)	0.8374 (3)	0.0532 (7)	
H3	0.6209	0.0739	0.8948	0.064*	
C4	0.4298 (5)	0.10753 (12)	0.7306 (3)	0.0496 (6)	
C5	0.4169 (6)	0.15329 (11)	0.6493 (4)	0.0489 (6)	
C6	0.1188 (6)	0.09843 (13)	0.5717 (4)	0.0579 (7)	
C7	0.1361 (6)	0.18683 (13)	0.4530 (4)	0.0621 (8)	
H7A	0.1349	0.2207	0.5011	0.075*	
H7B	−0.0331	0.1784	0.4274	0.075*	
C8	0.2846 (7)	0.19138 (15)	0.3195 (4)	0.0706 (9)	
H8	0.2371	0.2173	0.2548	0.090 (12)*	
C9	0.4653 (9)	0.1641 (3)	0.2855 (6)	0.0939 (15)	
H9A	0.5201	0.1376	0.3466	0.113*	
H9B	0.5461	0.1701	0.1989	0.113*	
C10	0.1712 (7)	0.02523 (13)	0.7497 (4)	0.0694 (9)	
H10A	0.0091	0.0156	0.7137	0.083*	
H10B	0.1562	0.0311	0.8522	0.083*	
C11	0.3410 (8)	−0.01892 (15)	0.7256 (5)	0.0771 (10)	
H11	0.3027	−0.0503	0.7717	0.129 (19)*	
C12	0.5362 (7)	−0.01882 (17)	0.6479 (6)	0.0832 (12)	
H12A	0.5828	0.0116	0.5995	0.100*	
H12B	0.6313	−0.0491	0.6400	0.100*	

### Atomic displacement parameters (Å^2^)

**Table:**

	*U*^11^	*U*^22^	*U*^33^	*U*^12^	*U*^13^	*U*^23^
Br1	0.05433 (18)	0.1026 (3)	0.0649 (2)	0.00262 (14)	−0.01189 (13)	−0.0127 (2)
N1	0.0564 (13)	0.0460 (14)	0.0731 (17)	−0.0070 (11)	−0.0083 (14)	0.0032 (13)
N2	0.0478 (13)	0.0545 (14)	0.0599 (15)	−0.0015 (10)	−0.0090 (11)	0.0029 (10)
N3	0.0493 (13)	0.0476 (13)	0.0686 (15)	−0.0089 (10)	−0.0042 (12)	0.0025 (11)
O1	0.0564 (12)	0.0847 (15)	0.0781 (16)	−0.0178 (10)	−0.0148 (16)	−0.0040 (16)
C1	0.0412 (14)	0.0642 (18)	0.0489 (15)	0.0007 (13)	−0.0024 (12)	−0.0096 (13)
C2	0.0507 (16)	0.0556 (17)	0.071 (2)	−0.0074 (13)	−0.0041 (15)	−0.0080 (15)
C3	0.0489 (15)	0.0567 (17)	0.0539 (16)	0.0026 (13)	0.0034 (14)	0.0046 (13)
C4	0.0416 (14)	0.0549 (17)	0.0522 (16)	−0.0013 (11)	0.0038 (12)	−0.0024 (13)
C5	0.0446 (14)	0.0454 (16)	0.0566 (17)	0.0003 (11)	−0.0005 (14)	−0.0055 (13)
C6	0.0531 (17)	0.0630 (18)	0.0575 (17)	−0.0051 (14)	−0.0016 (15)	−0.0051 (15)
C7	0.0550 (17)	0.0651 (19)	0.0662 (19)	0.0019 (13)	−0.0108 (15)	0.0016 (15)
C8	0.077 (2)	0.083 (2)	0.0523 (17)	−0.0094 (19)	−0.0090 (18)	0.0031 (17)
C9	0.092 (4)	0.119 (4)	0.071 (3)	0.003 (2)	0.017 (2)	−0.005 (3)
C10	0.065 (2)	0.062 (2)	0.081 (2)	−0.0164 (15)	0.0003 (19)	0.0097 (17)
C11	0.089 (3)	0.056 (2)	0.086 (3)	−0.0155 (18)	0.004 (2)	0.0058 (18)
C12	0.086 (3)	0.066 (3)	0.098 (3)	−0.0118 (17)	0.008 (2)	−0.014 (2)

### Geometric parameters (Å, °)

**Table:**

Br1—C1	1.896 (3)	C4—C5	1.390 (4)
N1—C5	1.328 (4)	C7—C8	1.485 (5)
N1—C2	1.345 (4)	C7—H7A	0.9700
N2—C5	1.383 (4)	C7—H7B	0.9700
N2—C6	1.388 (4)	C8—C9	1.240 (6)
N2—C7	1.444 (4)	C8—H8	0.9300
N3—C4	1.376 (4)	C9—H9A	0.9300
N3—C6	1.381 (4)	C9—H9B	0.9300
N3—C10	1.461 (4)	C10—C11	1.467 (6)
O1—C6	1.218 (4)	C10—H10A	0.9700
C1—C2	1.359 (4)	C10—H10B	0.9700
C1—C3	1.404 (4)	C11—C12	1.281 (6)
C2—H2	0.9300	C11—H11	0.9300
C3—C4	1.375 (5)	C12—H12A	0.9300
C3—H3	0.9300	C12—H12B	0.9300
			
C5—N1—C2	113.5 (3)	N2—C7—C8	114.0 (3)
C5—N2—C6	108.7 (2)	N2—C7—H7A	108.7
C5—N2—C7	126.6 (2)	C8—C7—H7A	108.7
C6—N2—C7	124.5 (3)	N2—C7—H7B	108.7
C4—N3—C6	109.6 (3)	C8—C7—H7B	108.7
C4—N3—C10	125.6 (3)	H7A—C7—H7B	107.6
C6—N3—C10	123.9 (3)	C9—C8—C7	126.6 (4)
C2—C1—C3	122.2 (3)	C9—C8—H8	116.7
C2—C1—Br1	119.3 (2)	C7—C8—H8	116.7
C3—C1—Br1	118.5 (2)	C8—C9—H9A	120.0
N1—C2—C1	124.0 (3)	C8—C9—H9B	120.0
N1—C2—H2	118.0	H9A—C9—H9B	120.0
C1—C2—H2	118.0	N3—C10—C11	114.1 (3)
C4—C3—C1	114.4 (3)	N3—C10—H10A	108.7
C4—C3—H3	122.8	C11—C10—H10A	108.7
C1—C3—H3	122.8	N3—C10—H10B	108.7
C3—C4—N3	133.4 (3)	C11—C10—H10B	108.7
C3—C4—C5	119.3 (3)	H10A—C10—H10B	107.6
N3—C4—C5	107.3 (3)	C12—C11—C10	127.0 (4)
N1—C5—N2	125.5 (3)	C12—C11—H11	116.5
N1—C5—C4	126.7 (3)	C10—C11—H11	116.5
N2—C5—C4	107.8 (2)	C11—C12—H12A	120.0
O1—C6—N3	126.9 (3)	C11—C12—H12B	120.0
O1—C6—N2	126.4 (3)	H12A—C12—H12B	120.0
N3—C6—N2	106.6 (3)		
			
C5—N1—C2—C1	1.8 (5)	N3—C4—C5—N1	−179.7 (3)
C3—C1—C2—N1	−1.2 (5)	C3—C4—C5—N2	−179.1 (3)
Br1—C1—C2—N1	−179.5 (3)	N3—C4—C5—N2	0.5 (3)
C2—C1—C3—C4	0.2 (4)	C4—N3—C6—O1	−176.8 (4)
Br1—C1—C3—C4	178.6 (2)	C10—N3—C6—O1	−7.0 (5)
C1—C3—C4—N3	−179.4 (3)	C4—N3—C6—N2	0.7 (3)
C1—C3—C4—C5	0.0 (4)	C10—N3—C6—N2	170.5 (3)
C6—N3—C4—C3	178.7 (3)	C5—N2—C6—O1	177.2 (4)
C10—N3—C4—C3	9.2 (6)	C7—N2—C6—O1	1.4 (5)
C6—N3—C4—C5	−0.7 (3)	C5—N2—C6—N3	−0.4 (3)
C10—N3—C4—C5	−170.3 (3)	C7—N2—C6—N3	−176.1 (3)
C2—N1—C5—N2	178.2 (3)	C5—N2—C7—C8	81.6 (4)
C2—N1—C5—C4	−1.6 (5)	C6—N2—C7—C8	−103.5 (3)
C6—N2—C5—N1	−179.9 (3)	N2—C7—C8—C9	2.7 (6)
C7—N2—C5—N1	−4.3 (5)	C4—N3—C10—C11	−77.2 (4)
C6—N2—C5—C4	−0.1 (3)	C6—N3—C10—C11	114.7 (4)
C7—N2—C5—C4	175.5 (3)	N3—C10—C11—C12	−3.3 (6)
C3—C4—C5—N1	0.8 (5)		
